# Expression and Functional Characterization of *c-Fos* Gene in Chinese Fire-Bellied Newt *Cynops orientalis*

**DOI:** 10.3390/genes12020205

**Published:** 2021-01-30

**Authors:** Gang Ye, Yalong Feng, Zhaoxiang Mi, Du Wang, Shuai Lin, Fulin Chen, Jihong Cui, Yuan Yu

**Affiliations:** 1 Lab of Tissue Engineering, College of Life Sciences, Northwest University, Xi’an 710069, China; yegnwu@163.com (G.Y.); fengylnwu@163.com (Y.F.); mizxnwu@163.com (Z.M.); wangdnwu@163.com (D.W.); linshnwu@163.com (S.L.); chenfl@nwu.edu.cn (F.C.); cjh@nwu.edu.cn (J.C.); 2 Provincial Key Laboratory of Biotechnology of Shaanxi, Northwest University, Xi’an 710069, China; 3 Key Laboratory of Resource Biology and Biotechnology in Western China, Ministry of Education, School of Medicine, Northwest University, Xi’an 710069, China

**Keywords:** *Cynops orientalis*, *c-Fos*, expression patterns, limb regeneration, skin wound healing

## Abstract

*c-Fos* is an immediate-early gene that modulates cellular responses to a wide variety of stimuli and also plays an important role in tissue regeneration. However, the sequence and functions of *c-Fos* are still poorly understood in newts. This study describes the molecular cloning and characterization of the *c-Fos* gene (*Co-c-Fos*) of the Chinese fire-bellied newt, *Cynops orientalis*. The full-length *Co-c-Fos* cDNA sequence consists of a 1290 bp coding sequence that encoded 429 amino acids. The alignment and phylogenetic analyses reveal that the amino acid sequence of *Co-c-Fos* shared a conserved basic leucine zipper domain, including a nuclear localization sequence and a leucine heptad repeat. The *Co-c-Fos* mRNA is widely expressed in various tissues and is highly and uniformly expressed along the newt limb. After limb amputation, the expression of *Co-c-Fos* mRNA was immediately upregulated, but rapidly declined. However, the significant upregulation of Co-c-Fos protein expression was sustained for 24 h, overlapping with the wound healing stage of *C. orientalis* limb regeneration. To investigate if *Co-c-Fos* participate in newt wound healing, a skin wound healing model is employed. The results show that the treatment of T-5224, a selective c-Fos inhibitor, could largely impair the healing process of newt’s skin wound, as well as the injury-induced *matrix metalloproteinase-3* upregulation, which is fundamental to wound epithelium formation. These data suggest that *Co-c-Fos* might participate in wound healing by modulating the expression of its potential target gene *matrix metalloproteinase-3*. Our study provides important insights into mechanisms that are responsible for the initiation of newt limb regeneration.

## 1. Introduction

The ability to regenerate lost or damaged tissue will be of great benefit to those who suffer from organ failure or severe trauma. However, adult mammals have very limited capacities for tissue regeneration. Urodele amphibians, such as axolotls and newts, possess the remarkable ability to regenerate lost or damaged body parts, including the ocular tissues, tail, nervous system, and limbs throughout their entire lives [1]. Among the tissues and organs that can regenerate, the limb has been most extensively studied, making it an accepted model for revealing the mechanisms regulating tissue and organ regeneration [2,3]. Successful limb regeneration begins with wound healing, followed by limb bud formation and a final redevelopment phase [2]. Prior studies have noted the importance of matrix metalloproteinases (MMPs) in salamander limb regeneration [4,5,6]. Among these *MMP* genes, *MMP-3* (also known as *stromelysin-1*) is upregulated in migrating epithelial cells within hours of limb amputation and facilitates healing of the wound, which is fundamental to the following regenerative processes [6,7]. However, the mechanisms that are responsible for the early induction of *MMP-3* expression remain ambiguous.

Immediate-early genes (IEGs) comprise a group of genes that are rapidly and transiently activated following a variety of extracellular signals, such as growth factors, developmental signals, stress, and cell injury [8]. In recent years, researchers have focused on IEGs for their critical roles in cellular functions, including proliferation, differentiation, and metabolism [9]. Among other IEGs, *c-Fos* was originally isolated from mouse genomic DNA as a cellular counterpart of the *v-Fos* carried by the Finkel-Biskis-Jinkins osteosarcoma virus. Then, it was identified in humans [10], rats [11], chickens [12], and fish [13] in succession. *C-Fos* is expressed in various cell types and dimerizes with c-Jun to form the transcription factor, activator protein 1 (AP-1), which binds to the target gene DNA to regulate various cellular functions, including cell cycle progression [14], cell dedifferentiation [15], and extracellular matrix re-organization [16]. In mammalians, *c-Fos* plays an important role in skin wound healing [17], and regenerative processes of the central nervous system [18], bone [19], and liver [20]. Recently, it has been reported that the upregulation of *c-Fos* expression is critical to initiating a pro-regenerative response after spinal cord injury in the axolotl [21]. These pieces of evidence inspire us to investigate whether *c-Fos* plays a part in newt limb regeneration.

In this study, a full-length *c-Fos* cDNA was cloned from *Cynops orientalis*, the Chinese fire-bellied newts exhibiting an extraordinary capacity to regenerate their limbs after amputation [22,23,24], and the amino acid sequence of *C. orientalis c-Fos* (*Co-c-Fos*) gene was determined. Then the multiple sequence alignment and phylogenetic analysis were performed. Finally, we investigated the expression pattern of the newt *c-Fos* gene and its potential role in the process of newt limb regeneration.

## 2. Materials and Methods

### 2.1. Animals and Sample Collection

*C. orientalis* has a remarkable capacity of regenerating an entire limb post-amputation and is widely distributed in China. Adult newts used in this study were obtained from Dabie Mountain (Huanggang, China). Animals of 8–10 cm nose-to-tail length were maintained in individual aquaria at 22 ± 2 °C. All surgeries were performed under anesthesia with 0.1% Tricaine methanesulfonate (MS-222, Sigma-Aldrich, St. Louis, MI, USA), and all efforts were made to minimize suffering. The tissue samples, including the spleen, heart, intestine, dorsal root ganglia, limb, tail, liver, kidney, and stomach, were collected for RNA isolation immediately after the animals were sacrificed.

The newt limb regeneration experiment was performed as previously described [22,23]. Briefly, the newt limbs were amputated at the right upper arm, and then the protruding bones were trimmed. The regenerated limb samples were collected at 15 min, 30 min, 45 min, 1 h, 3 h, 6 h, 12 h, 1 d, 3 d, 7 d, 14 d, 30 d, 42 d post-amputation by cutting the arm 2 mm from the first amputation plane. At the same position, the limb tissue before amputation was used as control.

To establish a skin wound healing model, a circular piece of skin (1.5 mm in diameter) was carefully removed from the right upper arms, making sure not to damage the underlying muscle. Then, the samples were collected at 15 min, 30 min, 45 min, 1 h, 3 h, 6 h, 12 h, 1 d, and 3 d after wounding for RNA isolation. The limb tissue before wounding was used as the control. To visualize the wound closure, Fast Green (Sigma, USA) was used to stain damaged tissues and dead cells in the wound area [25].

### 2.2. PCR Amplification and Quantitative Real-Time PCR (qRT-PCR) Analysis

Total RNA was extracted from each sample using RNAiso Plus reagent (Takara, Beijing, China), and the reverse transcription was performed to generate cDNA using a Transcriptor First Strand cDNA Synthesis Kit (Roche, Basel, Switzerland). To obtain the complete coding sequence of *Co-c-Fos* gene, PCR amplification was performed with the following procedure: 5 min at 95 °C; 30 s at 95 °C, 45 s at 52 °C, 90 s at 72 °C for 35 cycles; 8 min at 72 °C. The qRT-PCR analysis was performed using SYBR Premix Ex Taq (TaKaRa, China) and a CFX96™ Real-Time PCR Detection System (Bio-Rad, Hercules, CA, USA) with the following parameters: 95 °C for 10 s; followed by 40 cycles at 95 °C for 5 s; and 60 °C for 30 s. *β-actin* housekeeping gene was selected as an internal reference for the analysis. For each cDNA sample, the target and reference genes were amplified independently in the same experimental run in triplicate. The relative gene expression level (amount of target normalized to the internal reference) was calculated using the 2^−ΔΔCt^ method. PCR primers used in this study were designed based on transcriptome sequencing, as shown in Table S1.

### 2.3. Bioinformatic Analyses

DNAMAN software (7.0) was used to analyze the obtained cDNA and deduced amino acid sequence of *Co-c-Fos*. The conserved domains of *Co-c-Fos* were predicted using the Conserved Domain Database tool in NCBI (https://www.ncbi.nlm.nih.gov/Structure/cdd/wrpsb.cgi) and the 2ZIP program [26] (http://2zip.molgen.mpg.de/). The predicted tertiary structure model of the basic leucine zipper (bZIP) domain of *Co-c-Fos* was constructed with the SWISS-MODEL workspace (https://www.expasy.org/resources/swiss-model). The ProtParam (http://www.expasy.org/tools/protparam.html) were used to calculate the molecular weight and isoelectric point (pI) of *Co-c-Fos*. Multiple sequence alignment was performed using CLUSTALW (https://www.genome.jp/tools-bin/clustalw) among species, and a consensus sequence was generated using criteria from MultAlin [27]: Uppercase is identity; lowercase is consensus level > 0.5; ! is anyone of IV; $ is anyone of LM; % is anyone of FY; # is anyone of NDQEBZ. Nuclear localization sequence (NLS) was predicated by using the NLSdb database (https://rostlab.org/services/nlsdb/). Through the neighbor-joining (NJ) method, a phylogenetic tree was constructed by MEGA 5.05.

### 2.4. Sectioning and Histological Staining

Samples were fixed with 4% paraformaldehyde for 24 h at 4 °C and then decalcified by 10% EDTA for 14 days. Samples were embedded in paraplast and sectioned at 4 μm. The slides were stained with Masson’s Trichrome using standard procedures.

### 2.5. Western Blot

Total protein and nuclear protein were isolated from the newt limbs (*n* = 6 per biological replicate) by using RIPA lysis buffer (Beyotime, Shanghai, China) and the Nuclear and Cytoplasmic Protein Extraction Kit (Beyotime, Shanghai, China), respectively. BCA protein assay (Thermo Fisher Scientific, Waltham, MA, USA) was used to quantify the protein contents. Proteins separated by SDS-PAGE were transferred onto PVDF membranes (Millipore, Burlington, MA, USA). The membranes were blocked with 5% non-fat milk and then incubated with primary antibodies against c-Fos (1:1000, Sangon, Shanghai, China), β-actin (1:1000, Abmart, Shanghai, China), or histone H3 (1:1000, Cell Signaling Technology, Danvers, MA, USA) at 4 °C overnight, followed by horseradish peroxidase (HRP)-conjugated goat anti-rabbit IgG (1:5000, Abmart, China) or goat anti-mouse IgG (1:5000, Abmart, China) at room temperature for 1 h. Finally, the membranes were incubated with enhanced chemiluminescence (ECL) reagent (Beyotime, Shanghai, China), and the grayscale values of the bands were analyzed by using ImageJ software.

### 2.6. Statistical Analyses

The data were expressed as means ± standard error (SE). The statistical differences were calculated using the Student’s *t*-test and one-way analysis of variance (ANOVA) in SPSS 17.0 software. Value at *p* < 0.05 was considered to be statistically significant.

## 3. Results

### 3.1. Co-c-Fos cDNA Cloning and Sequence Analyses

**Figure 1:**
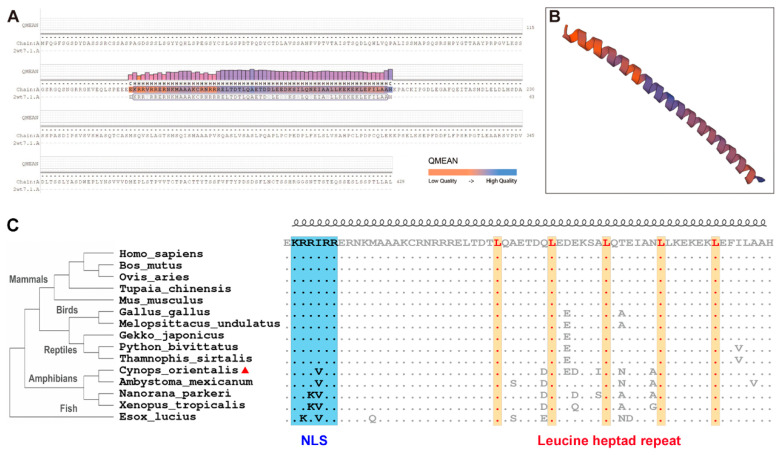
Protein structure homology modeling and phylogenetic reconstruction of *Co-c-Fos*. (**A**) Target–template alignment. The QMEAN Z-score indicates model quality and is visualized by using bar plots and a color scheme. (**B**) The predicted secondary structure exhibits continuous α-helices, which are displayed in a three-dimensional view. (**C**) Multiple sequence alignment and phylogenetic analysis of the bZIP domain among vertebrates. The reference sequence corresponds to the human bZIP domain. The predicted NLS (nuclear localization sequence) is shaped in blue, and the heptad-repeat leucines are highlighted in yellow.

The full-length cDNA sequence of *Co-c-Fos* was obtained and uploaded to NCBI, and the GenBank number is MG604921.1. The *Co-c-Fos* cDNA was 1602 bp and composed of an ORF of 1290 bp, a 185 bp 5′-untranslated region (UTR), and a 127 bp 3′-UTR. The predicted *Co-c-Fos* was a 429 amino acid protein with a bZIP domain, which shared a similar structure with that of the proto-oncogene protein *c-Fos* (SMTL ID: 2wt7.1.A, Figure 1A), and its predicted tertiary structure exhibited continuous α-helices (Figure 1B). The molecular weight of the predicted *Co-c-Fos* was estimated to be 45.86 kDa, and the pI was estimated at 4.67.

To assess the homology of c-Fos proteins among vertebrates, the multiple sequence alignment was performed. The result showed that the amino acid sequence of c-Fos was quite conserved among the selected species (Figure S1). However, *Co-c-Fos* exhibited higher similarity in amphibians (axolotl and frogs) than in other species, including reptiles, birds, mammals, and fish (Table S2). It was found that the *Co-c-Fos* protein shared a conserved bZIP domain, with a nuclear localization sequence (NLS) and a leucine heptad repeat included (Figure 1C). Interestingly, the predicted NLS of *Co-c-Fos* was KRRVRR, and the fourth valine residue was quite conserved among the lower vertebrates, including fish and amphibians, whereas it was KRRIRR for the higher vertebrates. A phylogenetic tree, rooted into *Esox lucius*, was used to investigate the evolutionary relationships of *Co-c-Fos*. As shown in Figure 1C, all *c-Fos* sequences were clustered into two clades. In one clade, the *c-Fos* sequences from mammals, birds, and reptiles were grouped, and the sequences from amphibians were clustered into the other clade, among which *C. orientalis* was clustered with the Mexican axolotl (*Ambystoma maculatum*). Our results indicated that the branch of *c-Fos* is classified according to the evolutionary positions of the species of fish, amphibians, reptiles, birds, and mammals, and the phylogenetic position of *Co-c-Fos* is consistent with the location of *C. orientalis*.

### 3.2. Distribution of Co-c-Fos mRNA Expression in Various Tissues of C. orientalis

**Figure 2:**
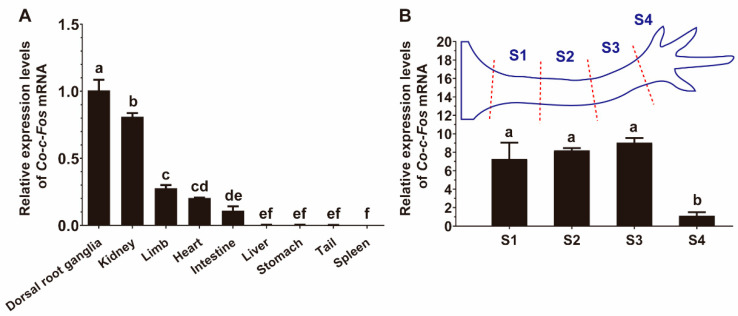
Tissue distribution of *Co-c-Fos* mRNA expression. (**A**) Relative mRNA expression levels of *Co-c-Fos* in various tissues of *Cynops orientalis.* The mRNA expression level of *Co-c-Fos* in dorsal root ganglia was set to one (*n* = 3). (**B**) Relative mRNA expression levels of *Co-c-Fos* in the four limb segments as illustrated in a cartoon. The mRNA expression level of *Co-c-Fos* in the S4 segment was set to one (*n* = 3). Data were analyzed using one-way ANOVA with Tukey test, and means with different letters indicated significant statistical difference (*p* < 0.05).

qRT-PCR results showed that the mRNA expression levels of *Co-c-Fos* varied vastly in different tissues (Figure 2A). The *Co-c-Fos* mRNA was strongly expressed in dorsal root ganglia and kidney; moderately in limb, heart, intestine; and extremely low in the other tissues. To investigate whether the positional pattern of *Co-c-Fos* gene expression exists along the newt limb, we tested the mRNA expression levels in different segments of a whole limb. As shown in Figure 2B, there was no significant difference in the expression levels of *Co-c-Fos* among the segments S1, S2, and S3, however, the expression level in the segment S4 was remarkably lower.

### 3.3. Expression Pattern of Co-c-Fos Gene during the Newt Limb Regeneration

**Figure 3:**
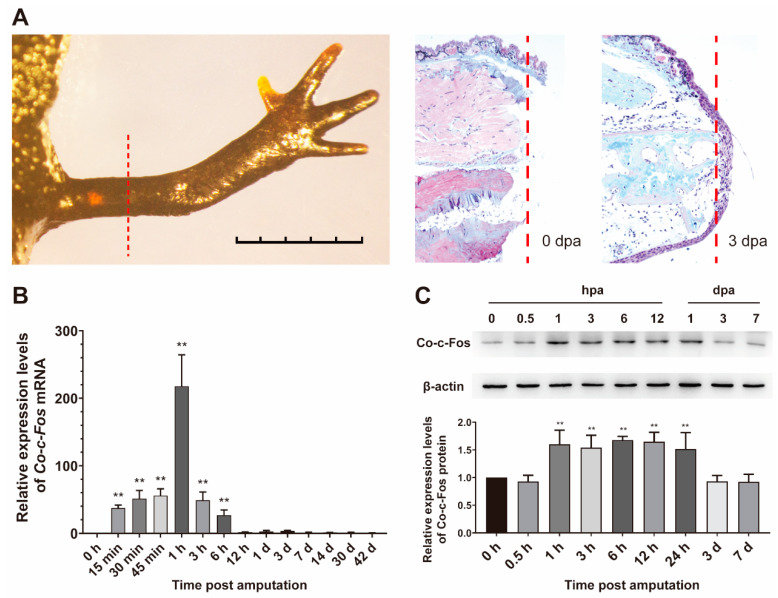
Expression pattern of *Co-c-Fos* gene during the newt limb regeneration. (**A**) The histological analysis of the limb regeneration with Masson’s trichrome at 0 and 3 days post-amputation. The red dashed line indicated the amputated position of the newt limb. (**B**) The mRNA expression pattern of *Co-c-Fos* during *C. orientalis* limb regeneration. The expression level of *Co-c-Fos* mRNA at 0 h was set to one (*n* = 3). (**C**) Western blot analysis of *Co-c-Fos* protein at the early stage of *C. orientalis* limb regeneration. The abundance ratio to β-actin was analyzed by densitometry. Data were analyzed using one-way ANOVA with the least-significant difference test. ** *p* < 0.05.

To better understand the expression pattern of the *Co-c-Fos* gene during *C. orientalis* limb regeneration, we collected and analyzed the regenerated limb samples at different time points post-amputation. These time points cover major morphological and physiological changes of limb regeneration throughout wound healing (within three days post-amputation, dpa), limb bud formation (7 dpa), blastema cell proliferation (14 dpa), chondrogenesis (30 dpa), up to digit formation (42 dpa) [22,24,28]. Histological analysis revealed that epidermal cells had covered the surface of the amputation site at 3 dpa (Figure 3A). qRT-PCR results showed that the expression of *Co-c-Fos* mRNA was immediately upregulated at 15 min post-amputation (*p* = 0.003) and then rapidly declined and returned to preamputation levels by 12 h post-amputation (hpa, *p* = 0.961) (Figure 3B). Also, the protein expression level of *Co-c-Fos* was detected by Western blot. It was found that the significant upregulation of *Co-c-Fos* protein was induced at 1 hpa (*p* = 0.001) and was sustained for 24 h (Figure 3C). Thus, the expression pattern of the *Co-c-Fos* gene, both at the mRNA and protein level, suggested its potential role in the early stage of newt limb regeneration.

### 3.4. Role of Co-c-Fos in the Process of the Newt Wound Healing

**Figure 4:**
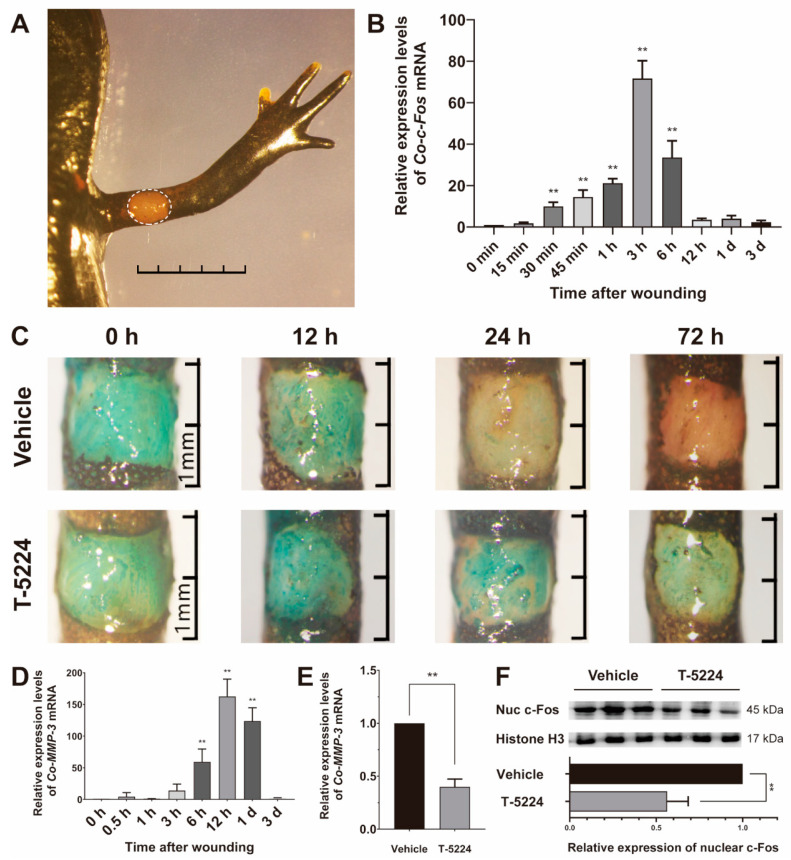
Role of *Co-c-Fos* in the process of the newt skin wound healing. (**A**) The newt skin wound healing model. The white outlines indicated the wound made in the limb of *C. orientalis.* Bar = 5 mm. (**B**) The mRNA expression pattern of *Co-c-Fos* mRNA during the newt skin wound healing. The mRNA expression level of *Co-c-Fos* at 0 min after wounding was set to one (*n* = 3). (**C**) Inhibitory effect of T-5224 on the newt skin wound healing visualized by Fast Green staining at 0, 12, 24, and 72 h after wounding. (**D**) The mRNA expression pattern of *Co-MMP-3* during the newt skin wound healing. The mRNA expression level of *Co-MMP-3* at 0 min after wounding was set to one (*n* = 3). (**E**) Inhibitory effect of T-5224 on the *Co-MMP-3* induction at 12 h after wounding. (**F**) Inhibitory effect of T-5224 on the nuclear accumulation of *Co-c-Fos* protein. The abundance ratio to Histone H3 was analyzed by densitometry. Data were analyzed using one-way ANOVA with the least-significant difference test in (**B**,**D**) and Student *t*-test in (**E**,**F**). ** *p* < 0.05.

The first step of limb regeneration is the healing of the wound, which is fundamental to the following regenerative processes [2]. To better understand the role of *Co-c-Fos* in wound healing, a skin wound healing model was established in *C. orientalis* (Figure 4A), in which the rapid and transient induction of *Co-c-Fos* was determined (Figure 4B), showing a similar expression pattern as observed in newt limb regeneration. Furthermore, a Fast Green staining method was employed to quickly visualize the wound closure. The Fast Green staining results showed that the wound epidermis had partly covered the wound area at 24 h and completely covered the wound at 72 h after wounding. However, the healing process was largely impaired after treatment of T-5224, a selective inhibitor of *c-Fos* [29] (Figure 4C), indicating the important role of *Co-c-Fos* in the newt wound healing.

Previous studies have revealed that *MMP-3*, one potential target gene of *c-Fos* [30,31], plays a crucial part in wound epithelium formation during newt limb regeneration [6]. To clarify the underlying mechanism of *Co-c-Fos* on wound healing, we first examined the expression patterns of *Co-MMP-3* in the newt skin wound healing model. The expression of *Co-MMP-3* mRNA was significantly upregulated within hours of wounding, reaching a peak at 12 h after wounding (*p* < 0.001, Figure 4D). However, the maximum expression of *Co-MMP-3* was remarkably suppressed by T-5224 treatment at 300 mM (*p* < 0.001, Figure 4E), which effectively suppressed the nuclear accumulation of *Co-c-Fos* protein as manifested by Western blot analysis (*p* = 0.003, Figure 4F). Additionally, the inhibitory effect of T-5224 on *Co-MMP-3* expression was also determined in the newt limb regeneration model (Figure S2). Together these results suggested that the *c-Fos* mediated wound healing in *C. orientalis* might be dependent on *Co-MMP-3*.

## 4. Discussion

In the present study, we first cloned and characterized the *Co-c-Fos* cDNA. The deduced *Co-c-Fos* contained a typical bZIP domain present in other known *c-Fos* proteins. A conserved heptad leucine repeat within the bZIP domain resembles the leucine dimerization motif, which is essential for the dimerization with the Jun subunit to assemble the transcription factor AP-1 [32], thereby stimulating gene transcription of its target genes [29,33]. NLS is a highly conserved lysine- and arginine-rich peptide that is essential for proper nuclear localization of transcription factors [34]. However, the mutation of NLS (V141I) appears to take place in reptiles and fixes during evolution. Nevertheless, the V141I mutation does not seem to influence the nuclear targeting of *c-Fos* proteins; at least in our study, the nuclear accumulation of *Co-c-Fos* was detected (Figure 4F and Figure S2). Taken together, the highly conserved heptad leucine repeat and NLS could be necessary for *Co-c-Fos* to exert its biological functions.

Next, we profiled the tissue distribution and expression pattern of the *Co-c-Fos* gene. Previous studies have reported that *c-Fos* is expressed in many types of vertebrate tissues [35,36]. Consistently, *Co-c-Fos* was found widely, but differentially expressed in various tissues of *C. orientalis*. Additionally, *Co-c-Fos* was uniformly expressed in both the forelimb and upper limb (Figure 2B), making it probably not involved in determining the proximodistal identity of the newt limb, like Prod-1 [37].

In mammals, the *c-Fos* expression is normally short-lived; it peaks at 30 to 60 min after stimulation and falls to basal expression after 90 min [38]. In this study, significant upregulation of *Co-c-Fos* expression was also detected immediately after amputation. However, the duration of *Co-c-Fos* upregulation was much longer (up to 6 hpa). Interestingly, the abundance of *Co-c-Fos* protein did not begin to decline until 3 dpa (Figure 3C). The long-term induction of *c-Fos* is also reported in other salamander species, including *Notophthalmus viridescens* (up to 24 h post serum stimulation) [39], and the Mexican axolotl (1 day post spinal cord ablation) [21]. However, whether the duration of *c-Fo*s expression underlies differences in regenerative competence between species should be further investigated in a future study.

Nevertheless, our results demonstrated that the upregulation of the *Co-c-Fos* gene was temporally overlapped with the wound healing stage of *C. orientalis* limb regeneration. At this stage, the amputation plane is covered with wound epithelium to minimize tissue damage, infection, and inflammatory response [2]. Then, the wound epithelium forms a thicken structure called the apical epithelial cap (AEC, Figure 3A), which secretes various growth factors to aid in limb outgrowth [40]. Given the importance of wound healing in initiating limb regeneration, we further employed a skin wound model to investigate whether *Co-c-Fos* takes part in the process of wound healing. The novel benzophenone derivative T-5224, designed by 3D pharmacophore modeling based on the crystal structure of the bZIP domain [29], served as a potential drug to selectively inhibit *Co-c-Fos* activity. It was noted that T-5224 treatment could retard the process of *C. orientalis* wound healing (Figure 4C), as well as the nuclear accumulation of *Co-c-Fos* protein (Figure 4F and Figure S2), indicating that *Co-c-Fos* contributed at least partially to the skin wound healing. 

Surprisingly, T-5224 appeared to exert an inhibitory effect on the induction of *Co-MMP-3* expression (Figure 4E). Prior studies have noted the importance of *MMP-3* in the process of wound healing during salamander limb regeneration [6,7]. In mammalians, *MMP-3* expression is closely associated with cell migration [41,42,43], and notably, mice that lacked *MMP-3* exhibit impaired skin healing, due to inadequate wound contraction [44]. In accordance with the present results, previous studies have demonstrated that the *MMP-3* expression can be partially inhibited by a *c-Fos* inhibitor. Administration of T-5224 prevents arthritis in a mouse model by reducing the amount of *MMP-3* in vivo in sera and joints and in vitro in synovial cell and chondrocyte cultures [29,45]. T-5224 also significantly suppress IL-1β-induced *MMP-3* expression in human nucleus pulposus cells and a mouse explant culture model of intervertebral disc (IVD) degeneration [46]. Therefore, we speculate that *Co-c-Fos* might participate in wound healing by modulating the expression of its potential target gene *MMP-3*.

## Data Availability

All relevant data are within the article. The data that support the findings of this study are available from the corresponding author upon reasonable request.

## Supplementary Materials

The following are available online at https://www.mdpi.com/2073-4425/12/2/205/s1, Figure S1: Multiple sequence alignment of *c-Fos* amino acid sequences, Figure S2: Inhibitory effect of T-5224 on the *Co-MMP-3* induction in the newt limb regeneration model. Table S1: PCR primers used in this study, Table S2: *c-Fos* amino acid sequence identity comparison with other species.

## Abbreviations

**IEGs**	immediate-early genes
**AP-1**	activator protein 1
**bZIP**	basic leucine zipper
**NLS**	Nuclear localization sequence
**UTR**	untranslated region
**MMP**	matrix metalloproteinase
**AEC**	apical epithelial cap
**dpa**	days post-amputation
**hpa**	hours post-amputation

## References

[1] Brockes J.P., Kumar A. (2008). Comparative aspects of animal regeneration. Annu. Rev. Cell Dev. Biol..

[2] Yokoyama H. (2008). Initiation of limb regeneration: The critical steps for regenerative capacity. Dev. Growth Differ..

[3] Kragl M., Knapp D., Nacu E., Khattak S., Maden M., Epperlein H.H., Tanaka E.M. (2009). Cells keep a memory of their tissue origin during axolotl limb regeneration. Nature.

[4] Miyazaki K., Uchiyama K., Imokawa Y., Yoshizato K. (1996). Cloning and characterization of cdnas for matrix metalloproteinases of regenerating newt limbs. Proc. Natl. Acad. Sci. USA.

[5] Yang E., Gardiner D., Carlson M., Nugas C., Bryant S. (1999). Expression of *mmp-9* and related matrix metalloproteinase genes during axolotl limb regeneration. Dev. Dyn. Off. Publ. Am. Assoc. Anat..

[6] Vinarsky V., Atkinson D.L., Stevenson T.J., Keating M.T., Odelberg S.J. (2005). Normal newt limb regeneration requires matrix metalloproteinase function. Dev. Biol..

[7] Mescher A. (1976). Effects on adult newt limb regeneration of partial and complete skin flaps over the amputation surface. J. Exp. Zool..

[8] Niessen N., Balthazart J., Ball G.F., Charlier T. (2013). C-fos down-regulation inhibits testosterone-dependent male sexual behavior and the associated learning. Eur. J. Neurosci..

[9] Clark C.E., Hasan M., Bousso P. (2011). A role for the immediate early gene product c-fos in imprinting t cells with short-term memory for signal summation. PLoS ONE.

[10] Van Straaten F., Muller R., Curran T., Van Beveren C., Verma I.M. (1983). Complete nucleotide sequence of a human c-onc gene: Deduced amino acid sequence of the human c-fos protein. Proc. Natl. Acad. Sci. USA.

[11] Curran T., Gordon M.B., Rubino K.L., Sambucetti L.C. (1987). Isolation and characterization of the *c-fos* (rat) cdna and analysis of post-translational modification in vitro. Oncogene.

[12] Fujiwara K.T., Ashida K., Nishina H., Iba H., Miyajima N., Nishizawa M., Kawai S. (1987). The chicken *c-fos* gene: Cloning and nucleotide sequence analysis. J. Virol..

[13] Li Y., Kim I., Kim Y.J., Kim M.K., Yoon Y., Lee Y., Lee J. (2004). Cloning and sequence analysis of the self-fertilizing fish *Rivulus marmoratus* immediate early gene *c-fos*. Mar. Environ. Res..

[14] Wang S.H., Xu X., Xu F., Meng Y., Sun C., Shi L., Zhao E. (2016). Combined expression of c-jun, c-fos, and p53 improves estimation of prognosis in oral squamous cell carcinoma. Cancer Investig..

[15] Guo L., Sans M.D., Hou Y., Ernst S.A., Williams J.A. (2012). C-jun/ap-1 is required for cck-induced pancreatic acinar cell dedifferentiation and DNA synthesis in vitro. Am. J. Physiol. -Gastrointest. Liver Physiol..

[16] Florin L., Hummerich L., Dittrich B.T., Kokocinski F., Wrobel G., Gack S., Schorppkistner M., Werner S., Hahn M., Lichter P. (2004). Identification of novel ap-1 target genes in fibroblasts regulated during cutaneous wound healing. Oncogene.

[17] Cheng B., Liu H., Fu X., Sun T., Sheng Z. (2007). Recombinant human platelet-derived growth factor enhanced dermal wound healing by a pathway involving erk and c-fos in diabetic rats. J. Dermatol. Sci..

[18] Stern S., Knoll B. (2014). Cns axon regeneration inhibitors stimulate an immediate early gene response via map kinase-srf signaling. Mol. Brain.

[19] Ott C.E., Bauer S., Manke T., Ahrens S., Rodelsperger C., Grunhagen J., Kornak U., Duda G.N., Mundlos S., Robinson P.N. (2009). Mechanical strain of osteoblasts induces promiscuous and depolarization-induced immediate-early response genes. Bone.

[20] Hui T., Mizuguchi T., Sugiyama N., Avital I., Rozga J., Demetriou A.A. (2002). Immediate early genes and p21 regulation in liver of rats with acute hepatic failure. Am. J. Surg..

[21] Sabin K., Santosferreira T., Essig J., Rudasill S.E., Echeverri K. (2015). Dynamic membrane depolarization is an early regulator of ependymoglial cell response to spinal cord injury in axolotl. Dev. Biol..

[22] Tang J., Yu Y., Zheng H., Yin L., Sun M., Wang W., Cui J., Liu W., Xie X., Chen F. (2017). Itraq-based quantitative proteomic analysis of *Cynops orientalis* limb regeneration. BMC Genom..

[23] Yu Y., Tang J., Su J., Cui J., Xie X., Chen F. (2019). Integrative analysis of micrornaome, transcriptome, and proteome during the limb regeneration of *Cynops orientalis*. J. Proteome Res..

[24] Cui J., Zheng H., Zhang J., Jia L., Feng Y., Wang W., Li H., Chen F. (2017). Profiling of glycan alterations in regrowing limb tissues of *Cynops orientalis*. Wound Repair Regen..

[25] Weisenthal L.M., Marsden J.A., Dill P.L., Macaluso C.K. (1983). A novel dye exclusion method for testing in vitro chemosensitivity of human tumors. Cancer Res..

[26] Bornberg-Bauer E., Rivals E., Vingron M. (1998). Computational approaches to identify leucine zippers. Nucleic Acids Res..

[27] Corpet F. (1988). Multiple sequence alignment with hierarchical clustering. Nucleic Acids Res..

[28] Feng Y., Feng J., Zheng H., Wang W., Chen F., Yu Y., Cui J. (2018). Molecular cloning, characterization, and expression analysis of the three cysteine and glycine-rich protein genes in the chinese fire-bellied newt *Cynops orientalis*. Gene.

[29] Aikawa Y., Morimoto K., Yamamoto T., Chaki H., Hashiramoto A., Narita H., Hirono S., Shiozawa S. (2008). Treatment of arthritis with a selective inhibitor of c-fos/activator protein-1. Nat. Biotechnol..

[30] Reunanen N., Li S., Ahonen M., Foschi M., Han J., Kähäri V. (2002). Activation of p38 α mapk enhances collagenase-1 (matrix metalloproteinase (mmp)-1) and stromelysin-1 (mmp-3) expression by mrna stabilization. J. Biol. Chem..

[31] Kim J., Kim S., Noh E., Song H., Lee G., Kwon K., Lee Y. (2018). Reversine inhibits mmp-1 and mmp-3 expressions by suppressing of ros/mapk/ap-1 activation in uv-stimulated human keratinocytes and dermal fibroblasts. Exp Derm..

[32] Glover J.N., Harrison S.C. (1995). Crystal structure of the heterodimeric bzip transcription factor c-fos-c-jun bound to DNA. Nature.

[33] Karin M., Liu Z., Zandi E. (1997). Ap-1 function and regulation. Curr. Opin. Cell Biol..

[34] Dingwall C., Laskey R. (1991). Nuclear targeting sequences--a consensus?. Trends Biochem. Sci..

[35] Li H., Xie P., Li G., Hao L., Xiong Q. (2009). In vivo study on the effects of microcystin extracts on the expression profiles of proto-oncogenes (*c-fos*, *c-jun* and *c-myc*) in liver, kidney and testis of male wistar rats injected i.V. With toxins. Toxicon.

[36] Chatani K., Kawakami M., Weinstein J.N., Meller S.T., Gebhart G.F. (1995). Characterization of thermal hyperalgesia, c-fos expression, and alterations in neuropeptides after mechanical irritation of the dorsal root ganglion. Spine.

[37] Kumar A., Gates P.B., Brockes J.P. (2007). Positional identity of adult stem cells in salamander limb regeneration. Comptes Rendus Biol..

[38] Greenberg M.E., Ziff E.B. (1984). Stimulation of 3t3 cells induces transcription of the *c-fos* proto-oncogene. Nature.

[39] Yun M.H., Gates P.B., Brockes J.P. (2014). Sustained erk activation underlies reprogramming in regeneration-competent salamander cells and distinguishes them from their mammalian counterparts. Stem Cell Rep..

[40] Satoh A., Makanae A., Hirata A., Satou Y. (2011). Blastema induction in aneurogenic state and prrx-1 regulation by mmps and fgfs in ambystoma mexicanum limb regeneration. Dev. Biol..

[41] Huang S., Wu C., Chao D., Wu C., Li C., Chen G., Lan C. (2015). High-glucose-cultivated peripheral blood mononuclear cells impaired keratinocyte function via reduced il-22 expression: Implications on impaired diabetic wound healing. Exp. Derm..

[42] Ko C., Wang W., Wang S., Chu Y., Chang W., Wang J. (2014). Glycogen synthase kinase-3β-mediated ccaat/enhancer-binding protein delta phosphorylation in astrocytes promotes migration and activation of microglia/macrophages. Neurobiol. Aging.

[43] Wang S., Hsu J., Ko C., Chiu N., Kan W., Lai M., Wang J. (2016). Astrocytic ccaat/enhancer-binding protein delta contributes to glial scar formation and impairs functional recovery after spinal cord injury. Mol. Neurobiol..

[44] Bullard K., Lund L., Mudgett J., Mellin T., Hunt T., Murphy B., Ronan J., Werb Z., Banda M. (1999). Impaired wound contraction in stromelysin-1-deficient mice. Ann. Surg..

[45] Motomura H., Seki S., Shiozawa S., Aikawa Y., Nogami M., Kimura T. (2018). A selective c-fos/ap-1 inhibitor prevents cartilage destruction and subsequent osteophyte formation. Biochem. Biophys. Res. Commun..

[46] Makino H., Seki S., Yahara Y., Shiozawa S., Aikawa Y., Motomura H., Nogami M., Watanabe K., Sainoh T., Ito H. (2017). A selective inhibition of c-fos/activator protein-1 as a potential therapeutic target for intervertebral disc degeneration and associated pain. Sci. Rep..

