# Supplementary material for: Expression and Functional Characterization of c-Fos Gene in Chinese Fire-Bellied Newt Cynops orientalis
Source: Genes (Basel). 2021 Jan 30;12(2):205. doi: 10.3390/genes12020205 (PMC7912203; doi:10.3390/genes12020205)
Supplement: Supplementary file 1 [file genes-12-00205-s001.zip › Table_S1.pdf]

Table S1. PCR primers used in this study.

Primer	Sequence (5'-3')	Length (bp)	Purpose
Co-c-Fos_F<br>Co-c-Fos_R	GATTTCTGCACTGAGGA<br>ACACACACAAGAGAAGGCC	1602	PCR amplification
Co-c-Fos_qF<br>Co-c-Fos_qR	TCTGTCGTAGTGGACATGGA<br>GTAGGTGAAGACGAAGGAAGATG	93	qRT-PCR
Co-MMP-3_qF<br>Co-MMP-3_qR	GATGTTGATGGCATTCAAGCC<br>AGTTGGGTCACAGATGGATGG	144	qRT-PCR
β-actin_qF<br>β-actin_qR	CCGGATCATGTTTGAGACCTT<br>TCACCGGAATCCATCACAATAC	111	qRT-PCR
