# Supplementary material for: Expression and Functional Characterization of c-Fos Gene in Chinese Fire-Bellied Newt Cynops orientalis
Source: Genes (Basel). 2021 Jan 30;12(2):205. doi: 10.3390/genes12020205 (PMC7912203; doi:10.3390/genes12020205)
Supplement: Supplementary file 1 [file genes-12-00205-s001.zip › Table_S2.pdf]

Table S2. c-Fos amino acid sequence identity comparison with other species.

GenBank number	Speieces	Identity
MG604921.1	*Cynops orientalis*	100%
N/A*	*Ambystoma mexicanum*	82%
XP_018429844.1	*Nanorana parkeri*	62%
NP_001016200.1	*Xenopus tropicalis*	60%
XP_015276116.1	*Gekko japonicus*	58%
ELV09902.1	*Tupaia chinensis*	57%
XP_007425512.1	*Python bivittatus*	57%
XP_013917032.1	*Thamnophis sirtalis*	57%
XP_005148951.1	*Melopsittacus undulatus*	56%
NP_005243.1	*Homo sapiens*	54%
NP_034364.1	*Mus musculus*	54%
NP_001159654.1	*Ovis aries*	54%
ELR52625.1	*Bos mutus*	53%
XP_010862512.2	*Esox lucius*	52%
NP_990839.1	*Gallus gallus*	50%

* The sequence of the axolotl c-Fos homolog (AMEX60DD301011151.1) can be found in the AXOLOTL-OMICS database (https://www.axolotl-omics.org/).
