# Supplementary figures and images for: Expression and Functional Characterization of c-Fos Gene in Chinese Fire-Bellied Newt Cynops orientalis
Source: Genes (Basel). 2021 Jan 30;12(2):205. doi: 10.3390/genes12020205 (PMC7912203; doi:10.3390/genes12020205)

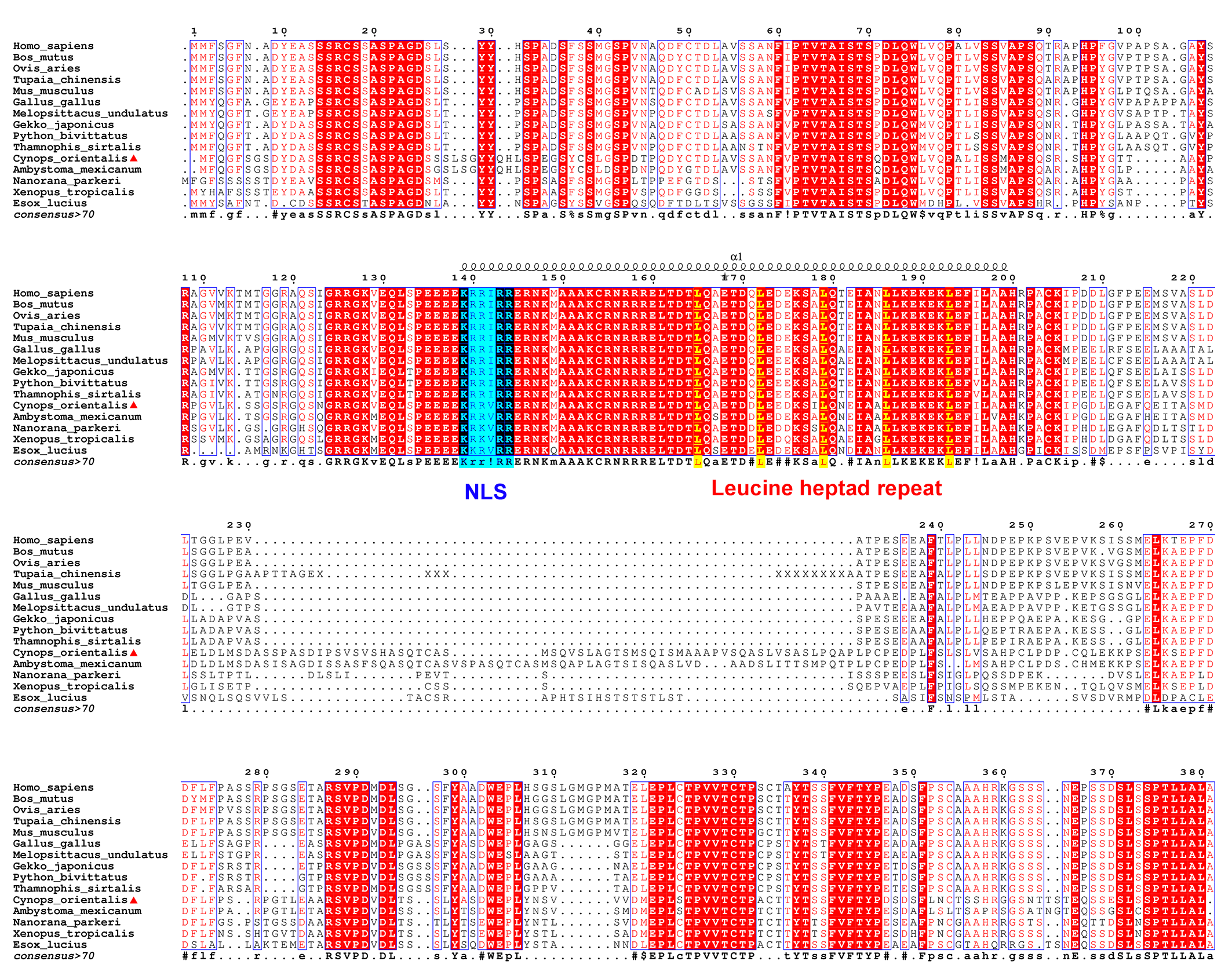

Supplement: Supplementary file 1 [file genes-12-00205-s001.zip › Figure_S1.tif]

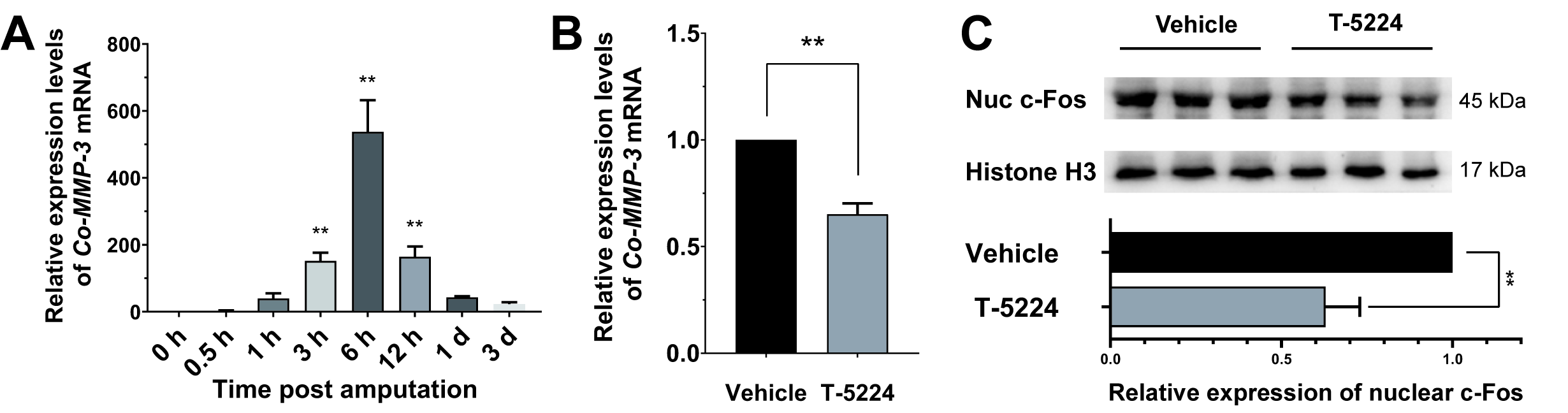

Supplement: Supplementary file 1 [file genes-12-00205-s001.zip › Figure_S2.tif]
